# Supplementary material for: PEX1 is essential for glycosome biogenesis and trypanosomatid parasite survival
Source: Front Cell Infect Microbiol. 2024 Mar 6;14:1274506. doi: 10.3389/fcimb.2024.1274506 (PMC10952002; doi:10.3389/fcimb.2024.1274506)
Supplement: Supplementary file 1 [file DataSheet_1.pdf]

Supplementary Figures:

A.

Organism	*T. brucei*	*T. cruzi*	*L. donovani*	*H. sapiens*	*S. cerevisiae*	*A. thaliana*	*D. melanogaster*
T. brucei		23.7%	16.0%	13.8%	13.3%	16.1%	13.0%
T. cruzi	17.1%		15.7%	12.6%	14.5%	14.5%	15.0%
L. donovani	9.4%	9.4%		15.5%	14.4%	16.3%	14.6%
H. sapiens	5.6%	7%	8.1%		16.4%	15.9%	12.6%
S. cerevisiae	5.9%	5.7%	6.7%	7.5%		14.3%	14.5%
A. thaliana	6.8%	7.2%	7.8%	6.9%	5.9%		14.1%
D. melanogaster	5.6%	5.9%	7.2%	4.8%	5.5%	5.8%	

% SIMILARITY

% IDENTITY

B.

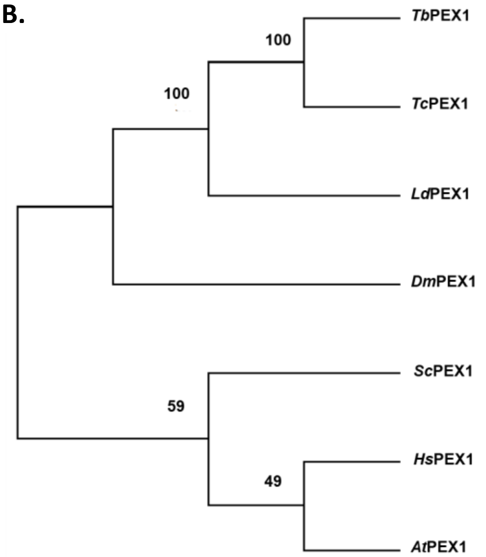

Supplementary Figure 1. A. PEX1 sequence identity and similarity matrix based on SIAS homology modelling **B.** Phylogenetic tree of PEX1 protein from different organisms (*Trypanosoma brucei* (*Tb*), *Trypanosoma cruzi* (*Tc*), *Saccharomyces cerevisiae* (*Sc*), *Homo sapiens* (*Hs*), *Leishmania donovani* (*Ld*), *Arabidopsis thaliana* (*At*), and *Drosophila melanogaster* (*Dm*)). The phylogenetic tree was constructed with the maximum likelihood (ML) method with 1000 bootstraps. The bootstrap values at each node represent the percentage of times the node was recovered in the 1000 bootstrap replicates. Bootstrap values of 70% or higher are generally considered to be good support for a node.

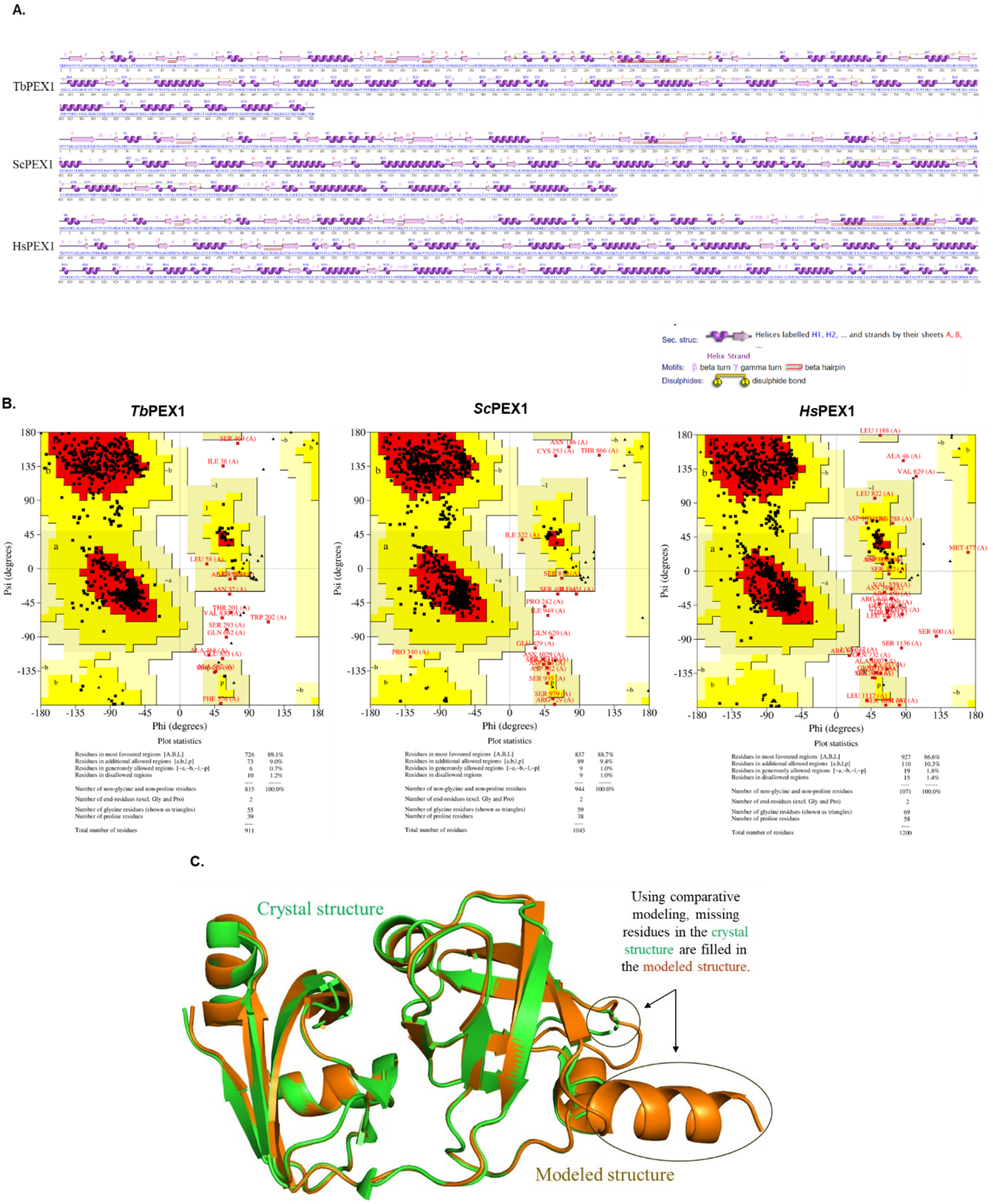

Supplementary Figure 2. A. 2D structure prediction of the putative PEX1 protein of *Trypanosoma brucei*, and the known PEX1 proteins of *Saccharomyces cerevisiae* and *Homo sapiens* **B.** Ramachandran plot for modelled PEX1 protein of *Trypanosoma brucei*, *Saccharomyces cerevisiae* and *Homo sapiens* obtained from Saves server of PROCHECK validation package. Less than 1.5% of residues were present in the Ramachandran plot's disallowed region **C.** Comparative modelling of the N-terminus of PEX1 3D structure showing the superimposition of the crystal structure of mouse PEX1 NTD (green) with the modelled structure of same protein (brown) with RMSD value of 0.29 Å. Same superimposition was also seen for the modelled structure of *Hs*PEX1 (95% sequence identity with mouse PEX1 NTD) with the crystal structure of mouse PEX1 NTD.

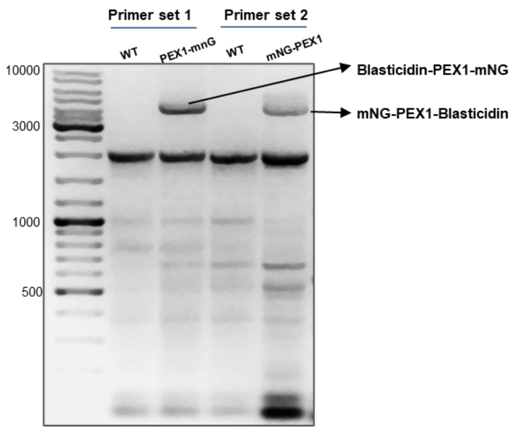

Supplementary Figure 3. PCR confirmation of endogenous tagging of *Tb*PEX1 with mNeonGreen in BSF trypanosomes using Primer set 1 (RE 7224-RE7225) and Primer set 2 (RE 7226-7227).

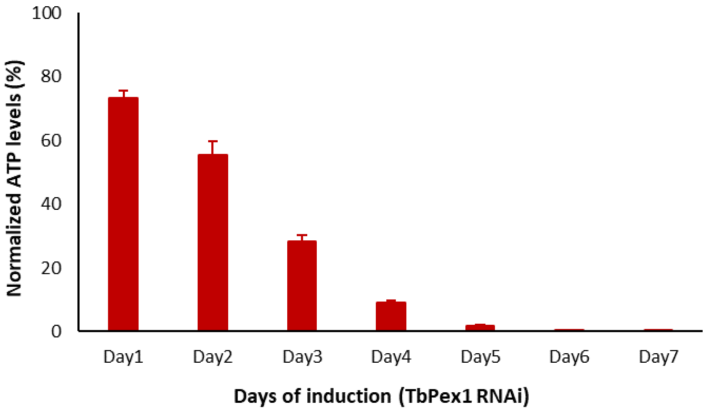

Supplementary Figure 4. Cell viability assessment of cells induced for PEX1 RNAi by CellTitre-Glo method. The viability measurement of RNAi induced cells (Tetracycline addition) were normalized to non-induced cells (DMSO treatment) in the respective days (day1 to day 7). Error bars represent the Standard deviation of the three biological replicates.

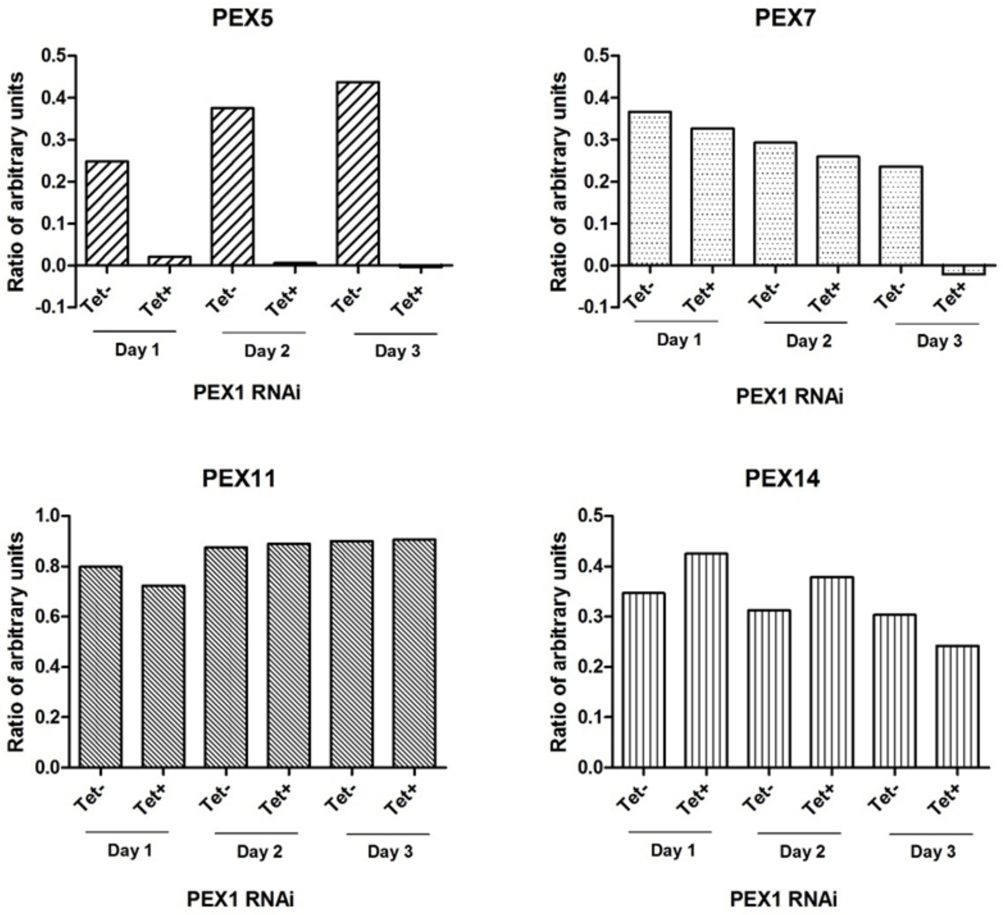

Supplementary Figure 5. Densitometric analysis of the glycosomal protein levels normalized over α-TbEnolase (loading control) for Day1 to Day3 of tetracycline treatment with PEX1 RNAi cells.

Supplementary Tables:

Table 1 Primers

No.	Primer name	Sequence (5’→3’)
1	RE7071	ACGATGTCGACAATGCAGCACAGCTCCTTTGAA
2	RE7072	ATAATGAGCTCTCACCGTTGGGTCAATTTCCTTCC
3	RE7073	ATCGAGTCGACCATGATGTCACGGACCGTTGAG
4	RE7074	ATAATACTAGTTCAATCAGCCACACGACCCGA
5	RE7224	AACAGGAAAGAATTCACCGCCTCTTCAGTAAGCAGGAAAAGAGCAGTACGAGGGA AGTCGGAAGGAAATTGACCCAACGGGGTTCTGGTAGTGGTTCC
6	RE7225	GAGCCCCAAGCCATACTGGAGAGGGCAGAGGAGTGGCTGACAGAAATTAACGAAT TGTAGAGTGTTTTGGTGTTACCACCCCAATTTGAGAGACCTGTGC
7	RE7226	TTTCTTCGTTCTCCAACCGATCTAGAGGGTCCCTTTATAAACGTAATTATTACTAGT GTAGCTCCAAAAAGAATAAGAGGGTATAATGCAGACCTGCTGC
8	RE7227	CGGATGAATTCATTTGACACCAAGACAAATGAGTCCGTTCGGGACGGGTCGATAGA AACTTCAAAGGAGCTGTGCTGCATACTACCCGATCCTGATCC
9	RE7323	ATGACAAAGCTTCATTGGCCAGAGTGAGCAGA
10	RE7324	ATGACAGGGCCCCCATACTCGCTGATGACGCT
11	RE7325	ATGACAGGATCCCATTGGCCAGAGTGAGCAGA
12	RE7326	ATGACAGGGCCCACGCTCCTCACAACGCTTGGA

Table 2 Strains and Plasmids

No.	Expression system	Construct	Primers	Restriction sites	Vector used for cloning
1	*Saccharomyces cerevisiae*	GAL4AD-*Tb*PEX1	RE7071-RE7072	SalI/SacI	PC86
2	*Saccharomyces cerevisiae*	GAL4BD-*Tb*PEX1	RE7071-RE7072	SalI/SacI	PC97
3	*Saccharomyces cerevisiae*	GAL4AD-*Tb*PEX6	RE7073-RE7074	SalI/SpeI	PC86
4	*Saccharomyces cerevisiae*	GAL4BD-*Tb*PEX6	RE7073-RE7074	SalI/SpeI	PC97
5	*Saccharomyces cerevisiae*	GAL4AD-*Sc*PEX1	Plasmid pIB1/31 (Birschmann et al., 2005)		
6	*Saccharomyces cerevisiae*	GAL4BD-*Sc*PEX6	Plasmid pBM21 (Birschmann et al., 2003)		
7	*Trypanosoma brucei*	*Tb*PEX1-mNG	RE7224-RE7225	-	pPOTV7
8	*Trypanosoma brucei*	mNG-*Tb*PEX1	RE7226-RE7227	-	pPOTV7
9	*Trypanosoma brucei*	Fragment I (*Tb*PEX1 Stem loop RNAi)	RE7323-RE7324	HindIII/ApaI	pHD1336
10	*Trypanosoma brucei*	Fragment II (*Tb*PEX1 Stem loop RNAi)	RE7325-RE7326	ApaI/BamHI	pHD1336
